# Flow as the mediating mechanism in marathon events: connecting event quality, motivation, and self-efficacy to perceived value and performance

**DOI:** 10.3389/fspor.2026.1765908

**Published:** 2026-04-01

**Authors:** Tianming Wu, Seungmo Kim, Taeyeon Oh

**Affiliations:** 1 Department of Sports and Health Sciences, Hong Kong Baptist University, Kowloon, Hong Kong SAR, China; 2 Department of Artificial Intelligence, Seoul AI School, aSSIST University, Seoul, Republic of Korea

**Keywords:** flow, antecedents, consequences, mediation, marathon, mass-participation event

## Abstract

**Objective:**

The purpose of the current study was to examine the relationships between key antecedent variables (event quality, motivation, and self-efficacy) and outcome variables (runners' perceived value and performance) through flow.

**Methods:**

Data were collected from 542 valid responses at the 2025 Beijing BCEG Miyun Marathon on-site. Partial Least Squares Structural Equation Modeling (PLS-SEM) and item parceling were employed to examine the proposed relationships in this study.

**Results:**

The results indicated that event quality, motivation, and self-efficacy significantly enhanced runners' flow, which, in turn, positively mediated the associations between event quality and perceived value and between self-efficacy and perceived performance.

**Conclusion:**

The findings confirmed that an evidence-based psychological framework can guide event organizers in designing optimal experiences that foster flow, thereby increasing participants' perceived economic and hedonic value. This research contributes to the sport management literature by refining the concept of flow in recreational contexts, distinguishing its experiential dimensions from its antecedents, and offering practical implications for the sustainable development of the mass-participation sporting events industry.

## Introduction

China's rapid economic expansion has been accompanied by intensifying work pressures and extended working hours, fostering a heightened public consciousness regarding health and well-being (1). In response, a growing segment of the population is actively adopting healthier lifestyles, with physical exercise becoming a central component of this shift (2). Running—a sport with a relatively low barrier to entry—has emerged as a particularly accessible and rapidly growing sport for years. According to the official website of the China Marathon, in 2024, a total of 749 road running events were held nationwide, attracting approximately 7.04 million participants (3). These events spanned across 261 cities in 31 provincial-level administrative regions nationwide, marking a substantial resurgence in post-pandemic participation.

Mass-participation running events can create valuable opportunities to inspire healthier lifestyles (4), enhance psychological well-being (5), improve city image (6), and attract tourism (7). It can also serve as a form of outdoor education, enhancing participants' experiential learning, self-awareness, and socialization (8). However, their success and long-term sustainability ultimately depend on participants' subjective experience. Therefore, event organizers should prioritize psychological design—clear goals, appropriate challenge, timely feedback, and social connection—over medals, souvenirs, and broad marketing to draw newcomers and encourage repeat participation. A deeper understanding of the participant's internal state during the event is paramount.

This current study utilized the concept of flow, “a state in which people are so involved in an activity that nothing else seems to matter; the experience is so enjoyable that people will continue to do it even at great cost, for the sheer sake of doing it” (9), to help event organizers prepare their following events with an evidence-based framework for understanding and designing peak psychological experiences that meaningfully increase participation in future events. In the context of running events, runners could cultivate flow during training, at the start line, and within pacing groups that balance challenge and skill. Flow could also be fostered along courses that provide clear progress cues and timely feedback and within environments energized by spectators, music, scenery, and social connection in the events. For example, new runners can turn anxiety into calm focus with good corrals and clear signs to guide them, while experienced runners experience the right level of challenge and better feedback from the course layout and how the race is organized.

The flow experience highlights how individuals become completely absorbed in an activity and lose a sense of their surroundings, a state that can occur in various situations, not just during physical activities. The diverse range of measurements and the growing interest from researchers have contributed to the development of new frameworks and the establishment of new research fields, such as EduFlow in education (10), WLOF in organization (11), EGameFlow in video games (12), and flow in online marketing (13).

While Csikszentmihalyi's nine-dimensional flow model provides a foundational framework for understanding optimal experience, its conceptual complexity and empirical ambiguities—particularly regarding construct overlap, internal dimension relationships, and inconsistent operationalization—pose significant challenges for application in specialized contexts such as recreational sporting events (14, 15). Scholars debate whether flow dimensions represent antecedents, experiences, or outcomes, with some emphasizing challenge-skill balance, clear goals, and immediate feedback as prerequisites (16, 17), while others view autotelic enjoyment as a driver of flow (18); further complicating matters, empirical studies often centered on challenge-skill balance due to high interdimensional correlations (19, 20), whereas alternative frameworks—such as the two-dimensional model of fluency and absorption (21)—or one dimensional measurement of psychological flow (22)—highlight the need for contextually adapted definitions. Moreover, flow's experiential and absorptive qualities overlap with constructs like engagement in PERMA (23) and experiential well-being (24), and it is not the sole state linked to peak performance in sports, as “clutch” states—marked by deliberate focus, effort, and situational awareness under pressure—also contribute to optimal outcomes (25, 26). Given the unresolved theoretical disputes and the scarcity of research applying flow theory to recreational event hosting, this study aims to address these gaps by examining how flow manifests among participants, thereby contributing novel insights to the sport management literature.

Despite the recognized importance of participant retention, the specific antecedents and consequences of the flow state within mass participatory sporting events remain insufficiently explored. The marathon event in the current study could offer a valuable opportunity to test refined flow frameworks that better distinguish between flow triggers, experiential dimensions, and outcomes. Therefore, the purpose of the current study was to empirically examine the relationships between key variables—event quality, motivation, and self-efficacy—and marathon runners' perceived value and performance, with flow positioned as the central mediating mechanism.

This study makes three key contributions to the literature. First, it extends flow theory from elite athletic contexts to the underexplored domain of recreational mass-participation events, offering ecological validity and practical relevance. Second, it addresses theoretical ambiguities in flow scholarship by empirically testing a mediation model that distinguishes between flow antecedents (event quality, motivation, self-efficacy) and consequences (perceived value, perceived performance). Third, methodologically, it employs PLS-SEM to capture the complex indirect effects through flow while leveraging post-event field data from a major Chinese marathon—a rapidly growing yet under-researched context. Collectively, this study contributes to both theory and practice by clarifying how flow can be intentionally cultivated through event design, thereby enhancing participant satisfaction, retention, and long-term engagement with healthy lifestyles.

### Conceptual framework

**Figure 1:**
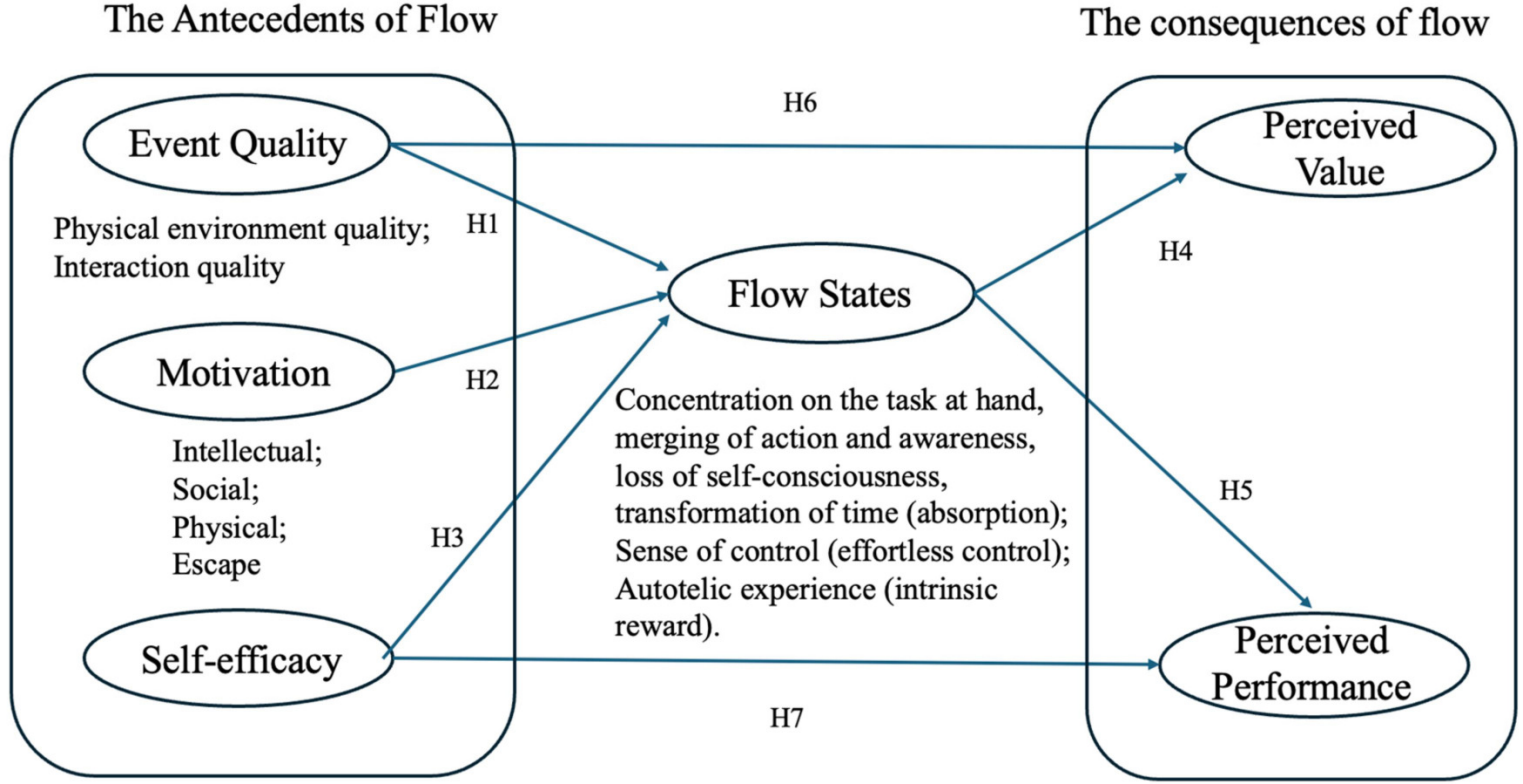
Theoretical framework.

Overall, the framework aims to clarify how these elements interact to facilitate the experience of flow. The framework encompasses the factors that contribute to achieving a flow state, comprising three main components. The antecedents of flow state include event quality (physical environment quality, interaction quality), motivation (intellectual, social, physical, and escapism dimensions), and self-efficacy. The flow state itself comprises six dimensions that define the flow experience in terms of absorption, effortlessness control, and intrinsic reward. The outcomes include perceived value and perceived performance. Figure 1 presents the study's conceptual framework.

### Flow state

The initial concept of psychological flow was introduced by Csikszentmihalyi, who identified nine dimensions characterizing an optimal psychological state of intrinsically motivating engagement (27). While this nine-dimensional framework has been foundational, contemporary flow scholarship increasingly distinguishes between structural antecedents that enable flow and the phenomenological core that constitutes the subjective experience itself (14, 28), reconsidering the validity and conflation of some subdimensions under different contexts (e.g., transformation of time, loss of self-conciseness) (29, 30), or developing new scales to reconceptualize the phenomenon of flow (21, 22), for a distinctive precision and improved measurement validity purpose. This conceptual refinement becomes particularly salient in the context of standardized recreational participatory events such as mass-participation marathons, where three traditionally included dimensions—challenge-skill balance, clear goals, and unambiguous feedback—are structurally embedded rather than emergent experiential states. Because recreational marathon participants operate within a highly constrained environment: the distance, markers, supplies, and wearable technologies that pre-specify challenge parameters and deliver continuous, objective feedback independent of subjective experience, it becomes both theoretically and methodologically appropriate to isolate the phenomenological essence of flow from its facilitating conditions. Consequently, measuring these dimensions as components of flow in this context risks capturing event design features rather than the participant's phenomenological state—a methodological conflation that threatens construct validity.

This study, therefore, advances theoretical precision by isolating flow's experiential essence—absorption (loss of self-consciousness, merging of action-awareness with concentrated attention), effortlessness with automaticity (sense of control without strain), and intrinsic reward (autotelic experience)—from its contextual prerequisites. While prior critiques of the nine-dimensional model have been largely general, we demonstrate how event standardization in mass-participation sport necessitates a domain-specific reconceptualization that treats certain dimensions as boundary conditions rather than experiential indicators. This approach not only resolves measurement ambiguity but also enables a more accurate assessment of flow's psychological mechanisms in standardized participatory contexts, focusing not only on the conditions enabling flow (addressed by event design, self-efficacy, and motivation) but also on the quality of the flow experience itself and beyond (perceived value and performance).

Absorption constitutes the foundational feature of flow, characterized by total concentration, a merging of action and awareness, and a temporary suspension of self-reflective thought (17, 31). In contexts like marathon running—where participants are prepared for optimal behavior, and the event structure minimizes cognitive load through standardized distance and immersive atmosphere—attentional resources are fully allocated to the present moment, enabling deep immersion that naturally diminishes awareness of self and time. This state aligns with the Transient Hypofrontality Hypothesis, which posits that during high-intensity physical tasks, extensive neural activation is required to run motor patterns, assimilate sensory inputs, and coordinate autonomic regulation, thereby reducing activity in the prefrontal cortex, leading to a reduced capacity for complex cognitive tasks during exercise (32). Consequently, loss of self-consciousness and time transformation are conceptualized as emergent features of absorption rather than standalone experiences: when attention is wholly invested in the external task, the default mode network becomes less active, and higher-order cognitive functions, such as self-reflection and time estimation, recede (33). Phenomenologically, absorption encompasses focused, undistracted attention that blocks external noise, action-awareness merging that dissolves the boundary between self and task, and a distortion of time perception that allows sustained engagement without fatigue or boredom (21, 34). Absorption is not merely one aspect, but the combination of concentration, the merging of action and awareness, loss of self-consciousness, and time transformation that could defining qualities of flow emerge.

Effortless control denotes a sense of mastery in which performance feels fluid, automatic, and free from strain, even during a relatively high physical exertion (35). Neuroscientific evidence reveals this state arises from efficient neural reorganization: increased activation in the left anterior inferior frontal gyrus (IFG) and putamen enhances goal-directed attention and reward processing, while simultaneous deactivation of the medial prefrontal cortex (MPFC) and amygdala suppresses self-referential thought and negative arousal (36, 37), supporting the effortless immersion and sense of control. The conflict-monitoring cognitive processes suggest that the brain is adaptive to cognitive control based on the detected level of conflict, thereby optimizing performance in the face of ongoing demands (38). It facilitates a transition from effortful, explicit control to intuitive, embodied fluency, freeing cognitive resources and enabling seamless task execution (39). Therefore, the transition from explicit, effortful control to more implicit, automatic processing is a hallmark of flow, freeing up cognitive resources and enhancing the feeling of mastery and control. Critically, this distinguishes flow from “clutch” performance, which involves deliberate, effortful focus under pressure (40). For recreational runners whose goals are often personal and enjoyable rather than professionally competitive, effortless control reflects the harmony between the body and the task, rather than the suppression of anxiety to achieve an outcome.

Finally, flow is inherently intrinsically rewarding (autotelic), marked by positive valence, optimal arousal, and a sense of enjoyment that arises from the activity itself (31). The strong link between attention and rewards indicates that sustained attention during a flow experience must be somewhat rewarding. These intrinsic rewards provide the internal satisfaction and emotional benefits that drive engagement and persistence without feeling overwhelmed or bored. This is evident from studies showing activation of midbrain reward structures (41) and increased dopamine production during flow (42). This unified experience is reinforced by synchronization theory, which explains flow as the harmonious coupling of attention networks with reward pathways, producing effortless control and intrinsic pleasure (43, 44). In the context of a recreational marathon where participants are numerous and extrinsic incentives like bonuses are scarce, this intrinsic reward could be a key driver of re-participation and psychological well-being (45).

Therefore, this study employs the six-dimension model of flow (46), excluding the subscales for challenge-skill balance, clear goals, and unambiguous feedback. because these excluded dimensions function as contextual prerequisites rather than experiential components of flow (17, 18). This model, by emphasizing the six key dimensions of flow (concentration, control, loss of self-consciousness, action-awareness merging, time transformation, and autotelic experience), better presents the experiential state of absorption, automaticity, and intrinsic reward, making it more contextually valid for recreational marathon runners.

### Antecedents of flow

#### Event quality

Event quality can be interpreted from two perspectives: service quality and event experience. Service quality highlights the tangible and interactive aspects that a service provider can offer to participants (47). It is often viewed as a multi-dimensional concept comprising service attributes that reflect participants' cognitive evaluations (48, 49). In the context of sporting events, researchers have emphasized that the environmental quality, interaction quality, and outcome quality—defined by the competitiveness of the games and player performance—are fundamental concerns for fans (50, 51). Although the outcome quality cannot be controlled by the organizer, it serves as a distinctive feature that separates the sporting event service quality from a broader context and makes sports competitions so captivating.

Compared to the ordinary customer experience, event experiences enable the creation of remarkable and unique moments among participants in a liminoid space (52, 53). This perception extends beyond cognitive evaluation and emphasizes the various affective responses that participation in events can evoke (54, 55). The affective responses represent the core appeal to participants, highlighting the subjectivity of feelings, reflecting consumer satisfaction with fantasy and enjoyment, and enhancing the formation of lasting and positive memories (56, 57). Accordingly, the evaluation of psychological states, such as emotion, immersion, and hedonism, is incorporated into the dimensions of event experience (58, 59). However, with the conceptual overlaps of immersion in flow (60) and hedonism in subjective well-being (61), the conceptualization and measurement of the event experience require further development. The current study adopted a service quality perspective and measured event quality based on the dimensions of physical environment and interaction quality, which play an inevitable and stimulating role in event experiences. It encompasses clear logistics and supportive staff, reduces distractions, and creates autotelic services, thereby directly facilitating the onset of a flow state by enhancing the overall experience. Thus, the hypothesis is as follows:

Hypothesis 1: Event quality enhances the flow state.

#### Motivation

Based on self-determination theory (62), three distinct motivational forces are postulated: a) intrinsic motivation (engaging in activities for their inherent pleasure and satisfaction), b) extrinsic motivation (engaging in activities for external rewards or to avoid punishment), and c) amotivation (lack of motivation or intention to act). Intrinsic motivation is positively correlated with the flow state in various sports contexts (63, 64). Participants who are intrinsically motivated often experience heightened concentration, peak performance, and positive emotions, all of which are characteristics of the flow state (65). Extrinsic motivation also plays a role in predicting flow. Factors such as competition and coaches' behaviors can impact athletes' perceptions of autonomy, competence, and relatedness, which are psychological mediators that affect flow states (66). Therefore, the motivation of intellectual, physical, social, and escape factors in the context of marathon event participation is expected to positively influence flow (67). Intellectual and physical represent participants' intrinsic motivation, driven by curiosity or learning for its own sake, and focused on health, fitness, appearance, or performance outcomes. In contrast, social and escape represent extrinsic motivation, aimed at gaining friendships or approval, and an avoidance motive to reduce stress or prevent negative feelings. Accordingly, the hypothesis is as follows:

Hypothesis 2: Motivation enhances the flow state.

#### Self-efficacy

Self-efficacy is the belief in one's own capability to plan and perform actions to attain a specific outcome, a concept rooted in Bandura's social cognitive theory (68). In the context of sport, it refers to an athlete's belief in their ability to execute the actions required to achieve specific performance outcomes. It is distinct from perceived competence, focusing specifically on process-oriented and task-related abilities rather than overall self-worth (69). Self-efficacy is shaped by mastery experiences, vicarious learning, social persuasion, and the management of physiological and emotional states (70, 71). These sources interact to influence athletes' beliefs about their capabilities in specific situations. People with high self-efficacy are more likely to persist through setbacks and perform better under pressure (72). Specifically, research consistently shows a positive, moderate relationship between self-efficacy and sports performance, with higher self-efficacy linked to better outcomes and improved stress management (73, 74). Individuals with higher self-efficacy are more likely to experience flow, as their perceptions of their abilities help them perceive challenges as surmountable and maintain the challenge-skill balance (42, 75, 76). Although studies also suggest a reciprocal effect where flow experiences can further enhance self-efficacy (77), this study assumes that attempting a high-challenge task, such as a marathon, requires the confidence to engage with it. High self-efficacy provides this confidence, motivating the individual to step into the stretch zone where the balance of challenge and skill meets and flow resides. Thus, the hypotheses are as follows:

Hypothesis 3: Self-efficacy has a positive impact on flow states.

### Consequences of flow

In recreational participatory sporting events, participants engage not merely to complete a physical challenge, but to derive intrinsic psychological benefits, such as enjoyment, emotional fulfillment, and a sense of personal accomplishment, that ultimately shape their overall experience. These subjective outcomes are captured through two constructs: perceived value and perceived performance. Drawing on microeconomic utility theory (78), perceived value reflects the participant's holistic assessment of gains (e.g., enjoyment, social connection, emotional fulfillment) relative to sacrifices (time, cost, and effort) (79, 80). Meanwhile, perceived performance is an internal, evaluative judgment of one's own capability and achievement during the event, independent of objective metrics (81).

Flow transforms the event into an autotelic experience, one that is valued for its own sake. Participants who enter flow report heightened enjoyment, reduced fatigue, and greater emotional satisfaction (21, 37), which directly elevates perceived value by increasing the gains compared to the losses. Simultaneously, the effortless control and seamless merging of action-awareness characteristic of flow foster a strong sense of competence, thereby boosting perceived performance, even if actual race time or placement remains insignificantly changed. In essence, flow acts as a psychological amplifier, intensifying positive affect and self-evaluation, making the experience feel more rewarding and personally successful. Moreover, service quality itself is often evaluated as part of the gains in the utility calculus: comfortable amenities and engaging post-race experiences contribute directly to satisfaction and emotional fulfillment, reinforcing the perception that the investment (time, money, effort) was worthwhile. Meanwhile, self-efficacy enhances perceived performance by influencing how participants interpret their effort and capabilities, leading to more positive self-evaluations. Together, these factors explain how psychological states and event design jointly shape satisfaction and self-assessment in recreational marathons. Thus, the hypotheses are as follows:

Hypothesis 4: Flow states positively impact perceived value.
Hypothesis 5: Flow states positively impact perceived performance.
Hypothesis 6: Event quality positively impacts perceived value.
Hypothesis 7: Self-efficacy positively impacts perceived performance.

## Methodology

### Instrumentation

The survey consisted of six demographic information questions: gender, age, educational background, monthly income, frequency of workouts, and participation group. The FSS-2 (46) for physical activities was utilized to measure antecedents and experiences of flow with 24 items under six dimensions of a) merging of action and awareness, b) concentration on the task at hand, c) sense of control, d) loss of self-consciousness, e) transformation of time, and f) autotelic experience. Participation motivation items were adopted from Filo, Funk (67) with four dimensions: a) intellectual, b) social, c) physical, and d) escape. The Endurance Sport Self-Efficacy Scale (82) with 11 items was adopted to measure self-efficacy. Event service quality with 8 items was adopted from Theodorakis, Kaplanidou (83). Perceived performance with 2 items was used from Du, Jordan (80), and perceived value Hyun and Jordan (79) with 3 items. The survey comprises 56 items, excluding demographics, all measured on 7-point Likert scales ranging from 1 (strongly disagree) to 7 (strongly agree).

To ensure the accurate translation of items from English to Chinese, a back-translation approach was employed (84). Bilingual researchers translated the scale from English into Chinese, followed by a separate pair of researchers who translated back to English to confirm the equivalence of items between the two languages. Afterward, the content validity of the initial items was evaluated by a panel of experts in sporting event management and university academic staff to enhance their clarity and readability (85).

### Data collection

The 2025 Beijing BCEG Miyun Marathon, scheduled for May 25th, 2025, is one of the elite-label marathon events certified by the International Association of Athletics Federations. This event features a full marathon, a half-marathon, and a mini-marathon (10 km), attracting approximately 45,000 runners from around the world, with 12,000 successfully registered in the event. Data collection was conducted on-site. A 16-person execution team was stationed near the finish line, equipped with electronic tablets, and approached runners to complete the electronic questionnaire. The researchers engaged with over 1,500 participants and successfully collected 610 questionnaires. After eliminating incomplete and duplicate responses, 542 valid questionnaires were retained.

Among the valid responses, 259 are male (47.79%), and 283 are female (52.21%). The number of participants in the Full Marathon is 209, which accounts for 38.56% of the total. The Half Marathon has 206 participants, making up 38.01%, while the Mini Marathon (10 km) has 127 participants, representing 23.43%. In terms of age distribution, 23.80% of the participants are between 26 and 35 years old, 44.28% are aged 36 to 45, and 20.30% fall within the 46 to 55 age range. Regarding their workout frequency, 8.30% of participants exercise 1 to 2 times a week, 54.24% work out 3 to 4 times a week, and 36.90% engage in physical activity 5 to 7 times per week.

### Data analysis

The relationships among the concepts were examined using Partial Least Squares Structural Equation Modeling (PLS-SEM). This method requires a smaller sample size compared to covariance-based Structural Equation Modeling (CB-SEM). Additionally, PLS-SEM focuses on prediction when estimating models and does not impose distributional assumptions on the data (86). All constructs with sub-dimensions are reflective. R 4.4.3 and SPSS were used for data analysis.

Given the model's complexity, item parceling was used to improve stability and interpretability (87). Before parceling, a Confirmatory Factor Analysis (CFA) of the individual items was conducted to confirm that the unidimensionality of each first-order construct was established. The balanced strategy was applied to constructs with more than three items, and the theory-oriented strategy was applied to constructs with sub-dimensions. After parceling, the CFA on parcels was conducted to check the overall fit and parcel reliabilities.

The overall assessment is as follows. First, based on a 1:5 to 1:10 sample size ratio, the minimum sample size is 280, which meets the criteria for further analysis (88). Second, the Common Method Bias (CMB) was assessed using the Harman single-factor test and the Variance Inflation Factors (VIF) in the inner model. If a single factor accounts for less than 50% of the total variance, or all VIFs in the inner model from a full collinearity test are 3.3 or lower, the model can be considered free of common method bias in the context of PLS-SEM (89). Third, the item parceling was applied as stated above. Lastly, the PLS-SEM was employed to test the hypotheses.

## Results

### Common method bias

Given that the data for all latent variables were collected from a single source (the marathon participants) at a single point in time, the potential for CMB required careful assessment. This study employed both procedural remedies during data collection and *post hoc* statistical tests to mitigate and assess the extent of potential bias (90). First, Participants were assured of the confidentiality and anonymity of their responses in the survey's introduction, encouraging honest answers. Crucially, data were collected from participants immediately after they crossed the finish line. This specific timing is essential, as the race experience, including their sense of flow, was highly salient and fresh in their minds, thereby minimizing retrospective and memory-related biases that can contribute to CMB. Second, Harman's single-factor test revealed that the first single factor accounted for 27.95% of the total variance, below the commonly accepted 50% threshold. Additionally, a full collinearity test was conducted by examining the VIF for all constructs in the structural model. The seven VIF values for the inner model paths ranged from 1.082 to 1.253, well below the most conservative threshold of 3.3 (91), indicating that collinearity is not an issue in the model. The combination of both procedural and statistical processes suggests that CMB is not a significant concern in this study.

### Item parceling validation

Prior to parcel formation, the CFA on the original item-level data yielded acceptable model fit (*P*-value of Chi-square < 0.01, CFI = 0.907, TLI = 0.901, RMSEA = 0.035, SRMR = 0.040) (92, 93), with all factor loadings exceeding 0.50 (range from 0.534 to 0.903) and Composite Reliability values (CR) ranging from 0.790 to 0.881 (88). Items were then parceled using different strategies: the 11-item self-efficacy scale was evenly distributed across three parcels, while multidimensional constructs were parceled within sub-dimensions, resulting in four parcels for motivation, two for event quality, and six for flow state. The parcel-level CFA demonstrated improved fit (*P*-value of Chi-square < 0.01, CFI = 0.972, TLI = 0.966, RMSEA = 0.042, SRMR = 0.032), with strong factor loadings (range from 0.767 to 0.938) and higher composite reliabilities (range from 0.838 to 0.941), confirming that parceling preserved construct validity while enhancing measurement precision.

### Measurement model

First, all the factor loadings exceed 0.708, indicating that the construct explains over 50 percent of the indicator's variance, thus demonstrating acceptable item reliability. Second, internal consistency reliability is assessed by Composite Reliability (CR) and Cronbach's alpha. All CR values fall within the 0.80 to 0.95 range, indicating satisfactory to good reliability with no redundancy (94, 95). It is acknowledged that the Cronbach's alpha for perceived performance was 0.627, which falls marginally below the recommended threshold of 0.7. Given that this value falls within the acceptable range for exploratory research (i.e., between 0.60 and 0.70) (86), and given the strong composite reliability (0.838) and satisfactory outer loadings for all items comprising this construct, Perceived Performance was retained in the model. All other constructs' Cronbach's alpha values exceed 0.7, indicating acceptable internal consistency. Third, the convergent validity of each construct is assessed using the Average Variance Extracted (AVE) metric. All values exceed 0.5, indicating that the construct explains at least 50% of the variance in its items (95). The results for reliability and convergent validity are presented in Table 1.

**Table 1:** Summary of measurement items, reliability, and factor loadings.

Constructs	Items	Factor loadings
Flow State (Cronbach's alpha = 0.924; CR = 0.941; AVE = 0.726)	Merging of action and awareness	0.869***
	Concentration on the task at hand	0.893***
	Sense of control	0.869***
	Loss of self-consciousness	0.814***
	Transformation of time	0.835***
	Autotelic experience	0.828***
Event Quality (Cronbach's alpha = 0.848; CR = 0.930; AVE = 0.868)	Physical environmental quality	0.925***
	Interaction quality	0.938***
Motivation (Cronbach's alpha = 0.826; CR = 0.885; AVE = 0.657)	Intellectual	0.767***
	Social	0.818***
	Physical	0.844***
	Escape	0.813***
Self-efficacy (Cronbach's alpha = 0.848; CR = 0.908; AVE = 0.766)	Parcel 1 (Item2,3,4,11)	0.874***
	Parcel 2 (Item5,6,7,10)	0.875***
	Parcel 3 (Item1,8,9)	0.878***
Perceived Performance (Cronbach's alpha = 0.627; CR = 0.838; AVE = 0.722)	PP1	0.787***
	PP2	0.909***
Perceived Value (Cronbach's alpha = 0.753; CR = 0.858; AVE = 0.669)	PV1	0.849***
	PV2	0.782***
	PV3	0.821***

All factor loadings are significant at the level of *p* = 0.001.

***: Significant at *p* < 0.001.

Discriminant validity of the constructs was established using the Fornell and Larcker criterion and the Heterotrait-Monotrait (HTMT) ratios for PLS-SEM (96). For the Fornell-Larcker criterion, the square root of the Average Variance Extracted (AVE) for each construct must exceed the estimated correlation between any pair of constructs. HTMT values above 0.9 indicate a lack of discriminant validity, while HTMT values below 0.85 suggest discriminant validity for constructs that are conceptually distinct. As shown in Table 2, both the HTMT and Fornell-Larcker criteria are met, indicating acceptable discriminant validity.

**Table 2:** Discriminant validity.

Construct	Event Quality	Flow State	Motivation	Perceived Value	Perceived Performance	Self-efficacy
Event Quality	0.932	0.274	0.272	0.221	0.147	0.335
Flow State	0.309	0.852	0.292	0.285	0.24	0.439
Motivation	0.325	0.331	0.811	0.281	0.212	0.379
Perceived Value	0.275	0.338	0.353	0.818	0.591	0.353
Perceived Performance	0.197	0.303	0.289	0.865	0.850	0.313
Self-efficacy	0.395	0.495	0.454	0.439	0.418	0.875

Values below the diagonal indicate the HTMT ratios between the latent constructs. Values on the diagonal represent the square root of the AVE values. Values above the diagonal reflect the estimated correlations among the latent constructs.

### Structural model

**Figure 2:**
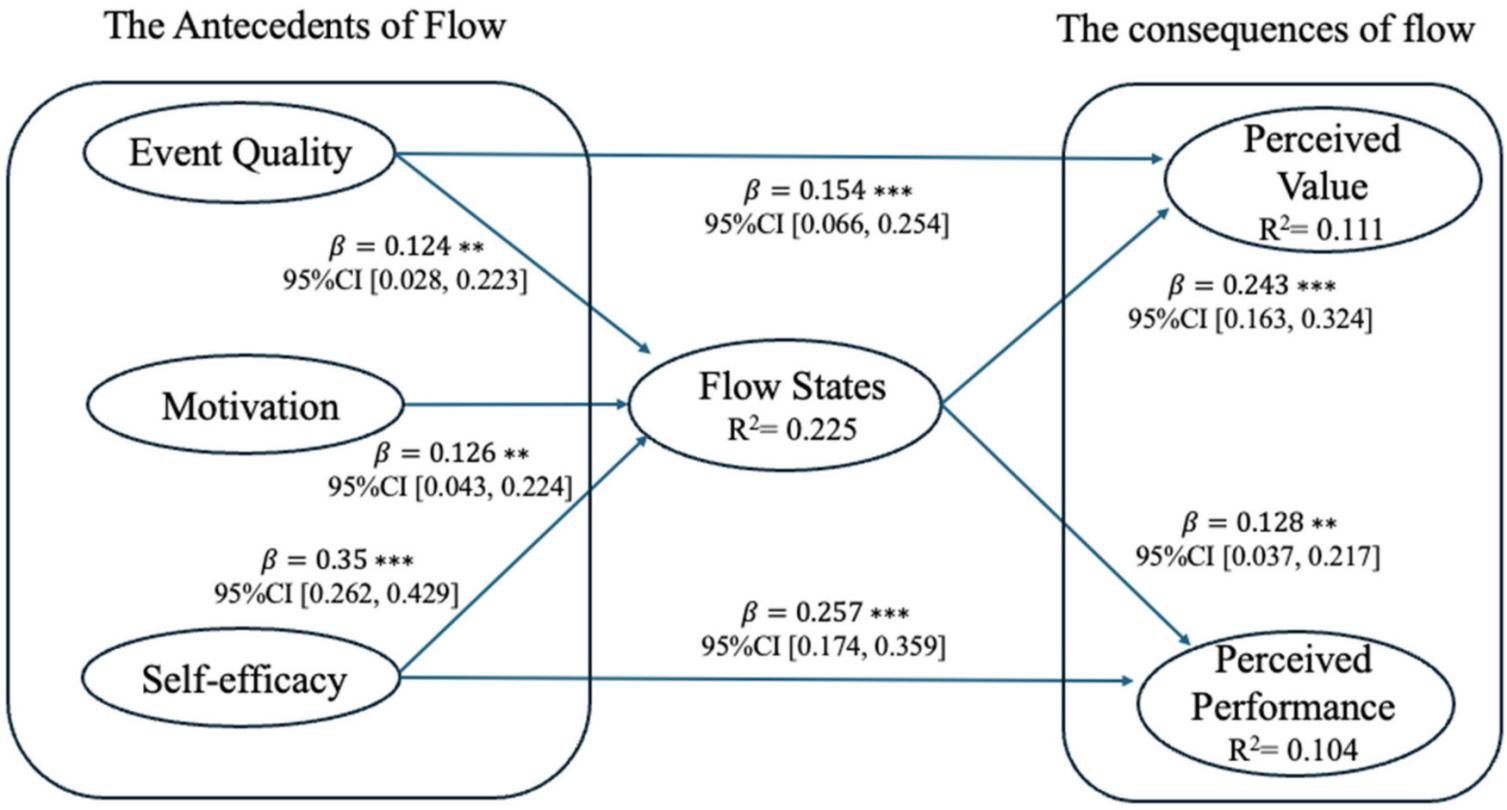
Structural model.

The structural model is assessed through three steps. First, the significance of the path coefficients and indirect effects was tested using a non-parametric bootstrapping procedure with 5,000 subsamples and bias-corrected 95% confidence intervals (97). As our hypotheses were directional, a one-tailed significance test was applied. All the hypotheses (Figure 2), indirect effects (Table 3), and total effects (Table 4) are significant. Second, the model's explanatory power was assessed via the coefficient of determination (R²). As shown in Figure 2, the R² values for all endogenous constructs were above the 0.10 threshold, indicating acceptable levels of variance explained in the dependent variables. Third, the model's predictive relevance was evaluated using the Q² metric, derived from a blindfolding procedure. While the Q² for flow state was moderate (0.208), the values for perceived value (0.090) and perceived performance (0.094) were small, suggesting only a small-to-modest predictive relevance for these particular endogenous variables (98).

**Table 3:** Indirect effect.

Specific indirect effects	Original sample	Sample mean	5.00%	95.00%	*P* values
Event Quality → Flow state → Perceived Value	0.03	0.03	0.009	0.052	0.010
Event Quality → Flow state → Perceived Performance	0.016	0.016	0.003	0.031	0.033
Motivation → Flow state → Perceived Value	0.031	0.032	0.013	0.055	0.008
Motivation → Flow state → Perceived Performance	0.016	0.017	0.004	0.034	0.039
Self-efficacy → Flow state → Perceived Value	0.085	0.087	0.055	0.122	0.001
Self-efficacy → Flow state → Perceived Performance	0.045	0.046	0.017	0.077	0.008

**Table 4:** Total effects.

Total effects	Original sample	Sample mean	5.00%	95.00%	*P* values
Event Quality → Perceived Value	0.184	0.186	0.109	0.265	0.001
Event Quality → Performance	0.016	0.016	0.003	0.031	0.033
Motivation → Perceived Value	0.031	0.032	0.051	0.21	0.008
Motivation → Performance	0.016	0.017	0.059	0.209	0.039
Self-efficacy → Perceived Value	0.085	0.087	0.055	0.122	0.001
Self-efficacy → Performance	0.302	0.305	0.229	0.378	0.001

## Discussion

This study developed and empirically validated a structural model demonstrating that flow arose from event quality, motivation, and self-efficacy and mediated their effects on runners' perceived value and performance. This study found that flow significantly mediated the relationship between event service quality and perceived value, suggesting that the influence of external event attributes largely depended on their ability to create an immersive psychological experience for participants. As previous studies in sport events found service quality as a key driver of satisfaction-related outcomes (50, 99), the findings of the present study also confirmed that event service quality shaped psychological outcomes by creating external conditions that promote immersive attendee experiences. These findings aligned with Mehrabian and Russell's (100) stimulus–organism–response model: event service quality functions as the stimulus, flow represents the elicited organismic state, and evaluations of value and performance are the responses, indicating that flow mediates the effect of service quality on these outcomes.

The findings of this study also revealed an important mediating role of flow between self-efficacy and perceived performance. Many empirical studies consistently found that the relationships between beliefs and actions could be mediated by intentions, goals, affect, and environmental factors in different contexts (101–103). This study extended social cognitive theory (68) by demonstrating that flow functions as the key mediator through which self-belief translates into performance-related benefits in marathon running. In other words, participants' self-efficacy could help create the conditions for entering a state of intense absorption and effortless control, which in turn led to more positive evaluations of their performance. These findings provided evidence of a dynamical interlink between social cognitive theory and Csikszentmihalyi's flow theory in high-performance recreation. This integration of cognitive and experiential paradigms offers a more comprehensive theoretical lens for understanding performance in participatory sports, acknowledging the path from belief to experience to evaluation.

### Theoretical contributions

The primary theoretical contribution of this study is the contextualization and empirical validation of the proposed framework, which echoes the Stimulus-Organism-Response (S-O-R) framework in the sport management literature by applying it to the phenomenon of flow in mass-participation sporting events (100). While flow research in sport is extensive, it has often failed to distinguish between the antecedents and consequences that trigger flow and the core experiential state itself in a practical recreational context. Our research addresses this gap by positioning the event environment and its characteristics as the Stimulus (S), the participant's subjective flow state as the internal Organism (O) response, and their subsequent value and performance perceptions as the final Response (R). This framework provides the necessary structure to clarify conceptual ambiguities previously highlighted in the literature (28).

A central theoretical stance of this paper is the reconceptualization of flow's structure specifically for the recreational, mass-participation context. We argue that three dimensions of Csikszentmihalyi's traditional model—challenge-skill balance, clear goals, and unambiguous feedback—function as antecedents (part of the Stimulus) rather than as components of the subjective experience itself. As stated above, in a marathon, the challenge (the distance), feedback (tracking devices), and the primary goal (to finish) are predefined environmental conditions. Therefore, our model focuses on the core phenomenological and autotelic dimensions that constitute the actual “Organism” state. This deliberate focus is further justified given documented validity concerns with some dimensions of the FSS-2 when including all nine dimensions, such as the transformation of time (104). By concentrating on the robust, core experiential components of absorption, effortless control, and intrinsic rewards, our study offers a parsimonious and valid measurement model for flow in this specific context.

This study empirically validated the experiential dimensions of flow in mass-participation events and revealed distinctions between competitive athletic performance and recreational sport engagement (40). By refining flow measurement specifically for recreational contexts and empirically distinguishing flow's experiential dimensions from its antecedents, this research offers a new insight into the performance-dominated paradigm in sport psychology by demonstrating that recreational participants prioritize experiential quality over competitive outcomes (105). It also provides empirical validation for the autotelic nature of recreational sport participation as a sustainable motivation mechanism, addressing calls for research on intrinsic motivation in recreational contexts (106). Additionally, it offers event organizers an evidence-based psychological framework for designing experiences that foster flow states, thereby enhancing participants' perceived economic and hedonic value (106).

More importantly, this study explicitly incorporates intrinsic reward as a defining characteristic of the autotelic experience in recreational sport participation—a theoretical contribution that distinguishes flow from mere immersion with fluency (21). This autotelic dimension represents a critical finding for the sustainable development of the mass-participation sporting events industry. Flow mediated the relationship between self-efficacy and perceived performance, suggesting that the intrinsic rewards of flow may enhance perceived performance even when objective performance metrics remain unchanged. We can speculate that when participants experience running as intrinsically rewarding, they will develop a lifelong love for physical activity, ensuring self-sustaining motivation. This result provides new insights into the literature on maintaining long-term participation in recreational sports and offers empirical support for applying flow theory to enhance well-being (107).

### Practical implications

The importance of flow in the context of a participatory sporting event lies in its ability to provide a high-level, optimal, and immersive mental state that cannot be experienced in daily life, as well as a form of active perception towards one's physical state.

First, our results demonstrate that flow significantly mediated the antecedents and consequences of flow, confirming that participants' event evaluations are fundamentally shaped by their psychological experience. High-level flow states prevent the two primary enemies of enjoyment: anxiety (when the challenge is too high) and boredom (when the challenge is too low) (108). For event organizers, creating absorption requires minimizing distractions through efficient registration, clear signage, and providing timely supplies, with visible volunteers, so participants can mentally and physically focus on the present moment. Effortless control and intrinsic rewards are key sources of satisfaction and personal achievement. To achieve this, it's important to align the event's challenges with the participants' skills. By designing multiple event difficulty levels, all participants can find their ideal challenge and experience a rewarding sense of flow.

Given that self-efficacy is the strongest antecedent of flow, organizers should prioritize capability-building interventions over generic motivational marketing to maximize participant immersion. Specific actions include providing structured pre-event training plans, hosting preparatory clinics, and offering course previews to bolster participants' belief in their ability to complete the challenge. While Event Quality has a weaker effect on Flow, it significantly impacts Perceived Value; thus, logistical excellence (e.g., friendly services, runners' packs with ample supplies) should be maintained as a hygiene factor to ensure financial satisfaction, even if it does not directly generate the psychological immersion required for Flow. The significant paths from motivation to flow suggest additional strategic levers. Marketing communications should segment audiences by their internal drivers: highlight timing services and personal bests for achievement-oriented runners, emphasize team registration and community atmosphere for socially-oriented participants, and focus on the transformative psychological experience for those seeking personal mastery.

Third, the relationships between flow and its outcomes provide a blueprint for post-event engagement. Because flow significantly predicts perceived value, organizers should communicate value in experiential rather than material terms, using participant testimonials and imagery that convey immersion and joy rather than focusing solely on finisher medals and T-shirts. Because flow significantly predicts perceived performance, organizers must recognize that for recreational participants, success is defined subjectively. A runner who achieves a personal peak experience feels they performed well, regardless of finish time. Event design should celebrate this intrinsic definition of performance through personalized finisher communications and progressive event series framed as opportunities to “find that feeling again”. In summary, flow is not a mysterious state but a psychological outcome that can be systematically cultivated by building self-efficacy pre-event, designing for immersion during the event, and celebrating experiential success post-event.

### Limitations and future research

While this study offers valuable theoretical and practical insights into the flow experience within recreational marathon running, its findings are bounded by several methodological limitations that warrant future research.

First, the reliance on a single, post-event survey leads several potential biases. The most immediate is recall bias, as participants were asked to retrospectively report their flow state immediately upon finishing the race. Although this timing minimizes delay, the experience itself may still be filtered through the lens of the event's outcome or the runners' overall fatigue. Furthermore, this design is subject to selection bias, which limits the generalizability of our findings. Data were collected exclusively from participants in a single large recreational running event in Beijing, China. Consequently, our examination of flow's antecedents and consequences is limited to the context of marathon running. Furthermore, the sample consisted solely of recreational runners, who may experience flow differently from professional athletes due to differing motivations, training regimens, and performance pressures. Future research should extend this line of inquiry to other sporting events, including spectator sports.

Second, one important statistical limitation concerns the model's predictive power. While the flow state demonstrated moderate predictive relevance, the Q² values for perceived value and perceived performance were small. This suggests that, although the model explains a significant portion of the variance in flow, its ability to accurately predict flow outcomes is limited. Other important factors not included in our model likely influence a runner's perception of value and performance. Future research should explore additional moderators or mediators, such as personal traits, emotions, or pre-race expectations, to build a more comprehensive predictive model. Another is the cross-sectional design. Given that all variables were assessed at a single time point using a cross-sectional design, the temporal precedence required to infer causal mediation could not be established (109). Thus, the mediation analysis should be interpreted as exploratory and aligned with theory, rather than as strong evidence of a causal mechanism. Future research should employ longitudinal designs, such as measuring antecedents' pre-race, flow during the race, and outcomes post-race, to provide stronger causal evidence for the proposed mediating mechanisms.

Lastly, CRs were strong across all constructs and most Cronbach's alphas were acceptable, but the alpha for perceived performance was slightly below the 0.70 benchmark. Although this value still falls within the acceptable range for exploratory research (86), future research should refine the measurement of this construct to further enhance its psychometric properties.

## Data Availability

The raw data supporting the conclusions of this article will be made available by the authors, without undue reservation.
